# Preoperative fasting protects aged-corpulent mice against renal ischemia-reperfusion injury

**DOI:** 10.1186/1753-6561-6-S3-P63

**Published:** 2012-06-01

**Authors:** Franny Jongbloed, Jan NM Uzermans, Sandra van den Engel, Harry van Steeg, Martijn ET Dollé, Ron WF de Bruin

**Affiliations:** 1 Departments of Surgery, Erasmus MC, University Medical Center, Rotterdam, 3015 GE, the Netherlands; 2 Laboratory of Health Protection Research, National Institute of Public Health and the Environment, Bilthoven, 3720 BA, the Netherlands

## Background

Oxidative stress (OS), the production of free oxygen radicals caused by for instance renal ischemia/reperfusion injury (IRI), results in age associated diseases and accelerated aging. We have shown that preoperative fasting in young-lean C57BL6 male mice protects against renal IRI [1]. Since human patients are usually older and suffer from (co)morbidities, we investigated the effects of preoperative fasting on OS induced by renal IRI in both female and male aged-corpulent mice in a F1 -FVB/C57BL6-hybrid background.

## Materials and methods

Male and female wild type littermates with an initial age of 72 weeks and average weight of 47.4 and 47.1 grams respectively were preoperatively giving either normal chow or were fasted for 72 hours followed by renal IRI. In the males, IRI was induced by clamping both renal pedicles for 37 minutes. In females, two ischemia times were applied: 37 and 60 minutes. Survival, body weight and wellbeing of the animals were monitored until day 28 postoperatively. P-values of <0.05 were considered significant.

## Results

Survival of male mice after 37 minutes of renal IRI was significantly better after 72 hours of fasting in comparison with the ad libitum fed mice (p=0.0171). All female mice survived in the first week after 37 minutes of IRI. When extended to 60 minutes of IRI, a significant difference between the two groups could be seen in favor of the fasted mice (p=0.0040). Body weight decreased in the first two weeks with normalization in the subsequent two weeks.

## Conclusions

Similar to young healthy male mice, preoperative fasting induces protection against renal IRI in both male and female aged mice. Old mice have a slower recovery of their body weight after surgery. These results suggest a general protective effect of dietary restriction against renal IRI, regardless of age, sex, body mass and genetic background. Therefore it may be applicable in older patients as well.
